# Effect of Ulinastatin in the Treatment of Postperative Cognitive Dysfunction: Review of Current Literature

**DOI:** 10.1155/2016/2571080

**Published:** 2016-08-14

**Authors:** Zheng-tao Lv, Jun-ming Huang, Jin-ming Zhang, Jia-ming Zhang, Jin-feng Guo, An-min Chen

**Affiliations:** Department of Orthopedics, Tongji Hospital, Tongji Medical College, Huazhong University of Science and Technology, Wuhan, Hubei 430030, China

## Abstract

*Background.* Ulinastatin, identified as a urinary trypsin inhibitor, has been widely used in patients with inflammatory disorders. However, little is known about its effect on postoperative cognitive dysfunction (POCD). The aim of our current work is to review the current body of literature.* Methods.* A systematic literature search in PubMed and EMBASE was performed to identify randomized controlled trials. Incidence of POCD, MMSE score, and laboratory indicators (IL-6, TNF-*α*, CRP, and S100*β*) were selected as outcomes.* Results.* Five RCTs involving 461 elderly patients that underwent surgical operations were identified. The meta-analysis suggested no statistical difference of incidence of POCD between ulinastatin and control groups on postoperative day 1; but ulinastatin could significantly decrease the incidence of POCD on postoperative day 3 and day 7 when compared with control treatment. Ulinastatin was effective in improving the MMSE score on day 1, day 3, and day 7 after operation. IL-6 and S100*β* concentrations were lower up to postoperative day 2. The incidences of postoperative complications in ulinastatin groups were lower than control.* Conclusion.* Ulinastatin administration was effective in treating early POCD (postoperative day 3 and day 7) and reducing IL-6 and S100*β* concentrations within two days after operations. Studies with larger-scale and rigorous design are urgently needed.

## 1. Introduction

Postoperative cognitive dysfunction (POCD) is a common situation that may occur after any sort of surgery and defined by a drop in cognitive domain on a set of neuropsychological tests from before to after surgery; it broadly refers to a “deterioration in cognition temporally associated with surgical operation” [1]. POCD impacts a wide variety of cognitive performance, for instance, memory, information processing, and executive function. The main subjective complaint is deterioration in memory, and some patients even find it hard to manage their jobs. The pathophysiology and etiology of POCD are relatively unknown, and higher age and preexisting cognitive impairment have been identified to date as risk factors [2–4].

Animal studies have suggested excessive neuroinflammation after surgery [5]; failure to effectively control inflammation may involve development of POCD [6, 7]. Using different preclinical models of noncentral nervous system surgery, neuroinflammation has been repeatedly associated with behavioral dysfunction and memory deficits. Upregulation of systemic inflammatory mediators and cytokines, including tumor necrosis factor-alpha (TNF-*α*), interleukin- (IL-) 1, interleukin- (IL-) 6, and C-reactive protein (CRP), has been shown to activate bone-marrow derived macrophages and contribute to the overall brain pathology after aseptic trauma [6–9]. Elevated serum levels of S100*β*, which indicate continuous tissue damage, have been reported to correlate with neurological deterioration after cardiac surgery and with poor likelihood of survival after hypoxia [10].

Ulinastatin not only have function in blocking the protease pathway, but have anti-inflammation proper in vitro as well [11]. In recent years, ulinastatin was wildly used in the treatment of a variety of severe diseases, such as disseminated intravascular pancreatitis, shock, and disseminated or diffuse intravascular coagulation [12, 13]. Some reports also support the role of protective in acute lung injury (ALI) and acute respiratory distress syndrome (ARDS) and multiple organ dysfunction syndrome (MODS) [14, 15]. The protective mechanism of ulinastatin may be attributed partly to the suppression of NF-Kappa B pathway [16] and mitogen-activated protein kinases (MAPKs) [17]. The suppression of aforementioned pathways could downregulate the production of proinflammatory cytokines, including TNF-*α*, IL-1, IL-6, and CRP [17–19].

Surgery is associated with a central neuroinflammatory response in humans. The inflammatory response may associate with incidence of POCD. Ulinastatin can significantly downregulate occurrence of inflammation. In addition, ulinastatin reported a possible protection of brain in gene level. Ulinastatin has certain therapeutic effects on cerebral ischemic reperfusion injury through upregulation expression of nerve growth factor and brain-derived neurotrophic factor and downregulation apoptosis of oligodendrocytes [20, 21]. However, little is known about its beneficial effect on POCD. Thus, the aim of our current systematic review and meta-analysis was to evaluate the clinical effect of ulinastatin on POCD based on randomized controlled trials (RCTs).

## 2. Methods and Materials

This systematic review was performed in accordance to the Preferred Reporting Items for Systematic Reviews and Meta-Analyses (PRISMA) guidelines [22].

### 2.1. Search Strategy

A comprehensive literature search was conducted in EMBASE and PubMed. Two electronic databases were searched from their inception date to the latest issue (January 2016), without language restriction. A combination of medical subject headings (MeSH) and free terms was used based on the specifications of each database. The search strategy for PubMed was as follow: (“urinastatin” [Supplementary Concept] or ulinastatin or UTI68 or urinary trypsin inhibitor or MR20 or miraclid) and ((Post-operative cognit^*∗*^  or Postoperative cognit^*∗*^  or POCD) and (surgery or operation) and (cognit^*∗*^  OR intelligence OR MMSE OR Mini Mental OR dementia OR Alzheim^*∗*^  OR mild cognitive impairment OR MCI)). The bibliographies of related systematic reviews and clinical guidelines were also searched. In addition, the reference section for each study was also searched.

### 2.2. Inclusion and Exclusion Criteria

We made our inclusion and exclusion criteria in adherence to the PICOS principle. P: subjects enrolled in our systematic review were patients undergoing surgical operations and no restriction on race, age, and gender was imposed; I: patients in ulinastatin groups were treated with ulinastatin intravenously before or/and after surgeries; C: control groups received a placebo administration of normal saline; the volume of normal saline must be the same as ulinastatin administration; O: the primary outcome measures included incidence of POCD and MMSE score; secondary outcomes were IL-6, TNF-*α*, CRP, and S100*β*; S: study design was restricted to RCTs. Case reports, case series, book chapters, and editorials were excluded.

### 2.3. Data Extraction

Two investigators (Zheng-tao Lv and Jun-ming Huang) screened each article independently and were blinded to the findings of the other reviewer. Following the prespecified inclusion criteria, two reviewers performed a rigorous screening to identify eligible articles. Data were collected from these selected articles using a standardized data collection sheet, which included first author, country, year of the publication, study design, cohort sizes, demographic characteristics of participants in different groups, details of intervention and control, and main outcomes.

Discrepancies between two reviewers was resolved through discussion until a general consensus could be reached. The third review author (Jin-ming Zhang) was sought for opinions if a consensus could not be reached.

### 2.4. Risk of Bias Assessment

To assess the risk of bias among our included studies, the Cochrane Collaboration's tool was utilized, which was based on seven items: random sequence generation, allocation concealment, blinding of participants and personnel, blinding of outcome assessment, incomplete outcome data, selective reporting, and other sources of bias [23]. Two reviewers (Zheng-tao Lv and Jun-ming Huang) judged the risk of bias among studies independently; the results were compared afterwards. In case of disagreements regarding the risk of bias judgement, discussion was conducted until a consensus was reached.

### 2.5. Data Synthesis

For the incidence of POCD, risk ratio (RR) and the associated 95% confidence interval (95% CI) were calculated using the Rev Man 5.3 (Copenhagen: the Nordic Cochrane Centre, the Cochrane Collaboration, 2014). Mean difference (MD) and the associated 95% CI were calculated for continuous variables using the same methodology. Before the combination of data from individual study, the chi-squared test and the Higgins *I* ^2^ test were used to assess the heterogeneity among studies (*P* > 0.1 and *I* ^2^ indicate acceptable heterogeneity). The fixed-effect model was used for statistical analysis if no obvious heterogeneity existed; random-effect model was employed if apparent heterogeneity existed.

Metaregression was performed to find the possible source of heterogeneity. Begg's rank correlation test and Egger's linear regression test were used to evaluate the publication bias if the number of included studies was greater than ten.

## 3. Results

### 3.1. Literature Search

**Figure 1:**
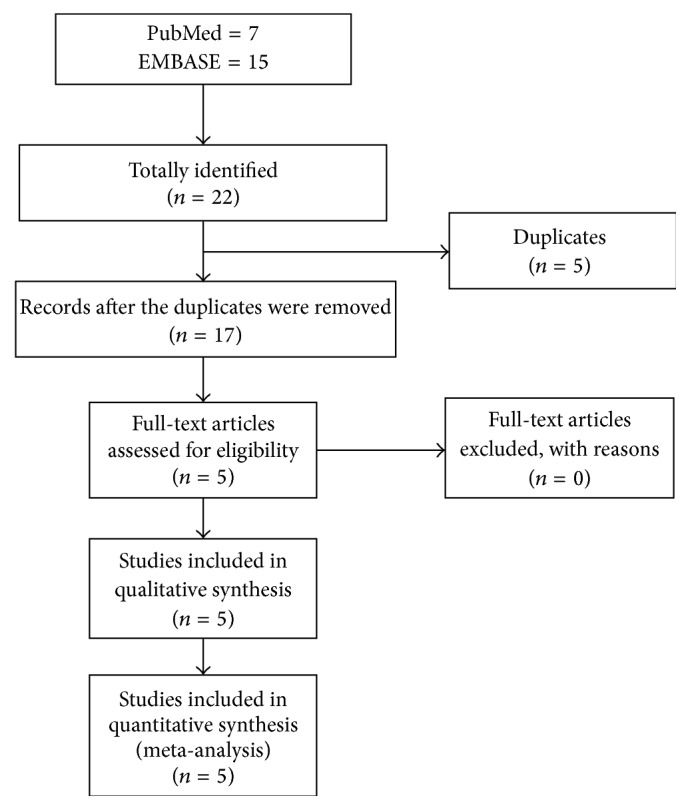
Flowchart of literature search and study selection.

An initial literature search yielded a total of 22 potential relevant citations including 7 from PubMed and 15 from EMBASE; 5 duplicates were deleted. At the stage of titles and abstracts screen, 12 articles were excluded because they were not related with ulinastatin in the treatment of POCD, and the remaining 5 articles were retrieved for a full-text review. Finally, 5 studies met our predetermined inclusion criteria (Figure 1).

### 3.2. Study Characteristics

All the included studies were conducted in China and published from 2010 to 2015; each study was undertaken in a single center. These five RCTs [24–28] included a total of 461 elderly patients that underwent surgical operations: 242 in the ulinastatin groups and 219 in the control groups. Three two-arm parallel RCTs [24, 25, 27] enrolled patients that underwent orthopedic operations; a prospective double-blind RCT [26] recruited participants scheduled for abdominal surgeries. Ge et al. [28] randomly assigned patients into three groups to investigate the clinical effect of ulinastatin on POCD: high dose ulinastatin group (16000 units/kg, i.v.), low dose ulinastatin group (8000 units/kg, i.v.), and control group (normal saline). The characteristics of included studies were summarized in Table 1.

### 3.3. Definition of POCD

 Xu and colleagues assessed the neuropsychological state of enrolled patients using a brief battery of neuropsychological test. A postoperative deficit in any test was defined by a decline of 20% or more from the preoperative value of that test; any patient demonstrating a deficit in 2 or more tests was considered as having POCD [29]. Ge and co-workers diagnosed POCD according to the same diagnostic criteria. The other three studies determined the incidence of POCD using MMSE scale. POCD was defined if the difference between post- and preoperative MMSE score was equal or larger than the standard deviation of preoperative MMSE scores [30].

### 3.4. Risk of Bias Assessment

**Figure 2:**
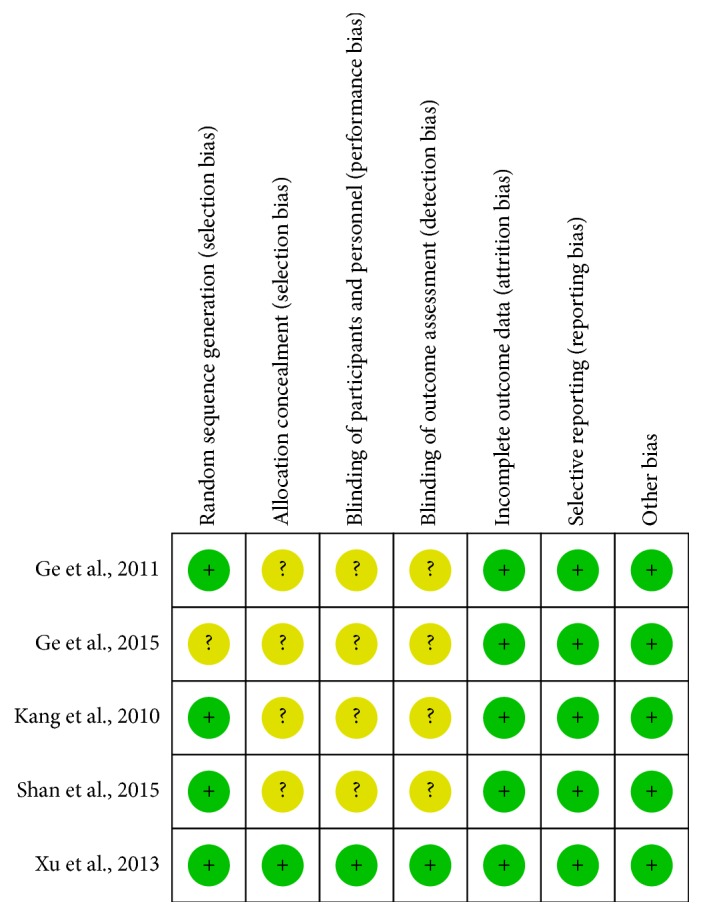
Risk of bias summary: review authors' judgements about each risk of bias item for each included study.

**Figure 3:**
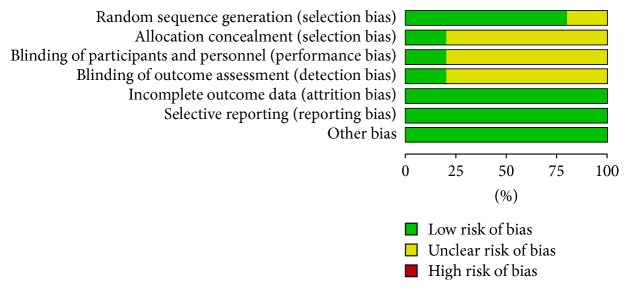
Risk of bias graph: review authors' judgements about each risk of bias item presented as percentages across all included studies.

All the studies included the suggested randomization; four studies reported the method of random sequence generation. Only one study reported procedure of allocation concealment and double-blinding: study drugs were prepared by the hospital pharmacy in identical containers marked with the name of the project, the investigator's name, and consecutive numbers. In this study, patients and investigators were blinded to the infusion. Two studies reported number of drop-outs; the reasons for withdraw in different groups were similar. When it comes to selective reporting bias, all studies were judged low risk of bias because we only included studies which reported incidence of POCD, MMSE score. The judgement of risk of bias was presented in Figures 2 and 3.

### 3.5. Incidence of POCD

**Figure 4:**
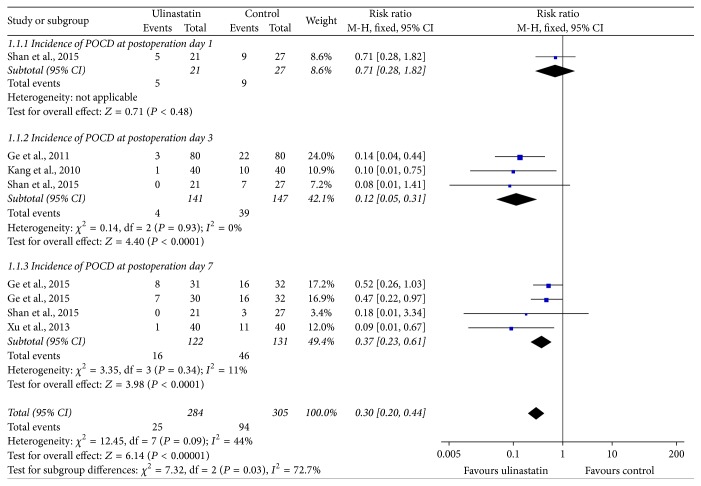
Forest plot of ulinastatin versus control: incidence of POCD.

All the included studies measured incidence of POCD as outcome assessment. As there was no obvious heterogeneity, fixed-effect model was utilized for statistical analysis. Subgroup analysis was conducted according to the different timing of neuropsychological tests. The meta-analysis showed no statistical difference of incidence of POCD between ulinastatin and control groups on postoperative day 1 (RR 0.71, 95% CI 0.28, 1.81; *P* = 0.48); but ulinastatin could significantly decrease the incidence of POCD on postoperative day 3 (RR 0.12, 95% CI 0.05, 0.31; *I* ^2^ = 0; *P* < 0.0001) and day 7 (RR 0.37, 95% CI 0.23, 0.61; *I* ^2^ = 11%; *P* < 0.0001) when compared with control treatment (Figure 4).

### 3.6. MMSE Score

**Figure 5:**
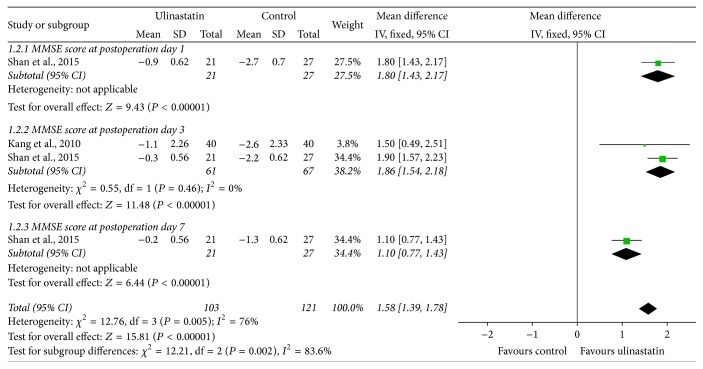
Forest plot of ulinastatin versus control: MMSE score.

Two studies [25, 27] measured MMSE score at different time point after surgeries. Subgroup analysis based on different timing of MMSE score measurement was conducted. The combination of data showed that ulinastatin could further improve the MMSE score on day 1 (RR 1.80, 95% CI 1.43, 2.17; *P* < 0.00001), day 3 (RR 1.86, 95% CI 1.54, 2.18; *P* < 0.00001), and day 7 (RR 1.10, 95% CI 0.77, 1.43; *P* < 0.00001) after operation (Figure 5).

### 3.7. Secondary Outcomes

In addition to the incidence of POCD and MMSE score, IL-6, TNF-*α*, CRP, and S100*β* concentrations were also measured by our included studies. Baseline similarities in ulinastatin and control groups were described in all the studies. Except for TNF-*α*, all these aforementioned laboratory indicators increased significantly within the first 24 postoperative hours. On day 3 after operation, there seemed to be no significant differences of IL-6, TNF-*α*, and S100*β*. IL-6 and S100*β* concentrations were significantly lower in ulinastatin groups than control groups from the end of operation to postoperative day 2; there were no significant differences of IL-6 and S100*β* on postoperative day 3. Regarding the changes of CRP and TNF-*α*, studies yielded conflicting results. The descriptions of secondary outcomes were listed in Table 2 in detail.

### 3.8. Other Outcomes

Two studies [24, 26] reported postoperative complications, such as nausea and vomiting. In the study conducted by Xu et al., the incidence of nausea and vomiting, arrhythmia, delirium, infection, allergic reaction, wound dehiscence, and myocardial ischemia was lower in ulinastatin group than control group, but the differences were not statistically significant. Ge and coworkers reported significantly lower incidence of deep vein thrombosis in ulinastatin group than control on the third day after hip joint replacement.

### 3.9. Publication Bias

**Figure 6:**
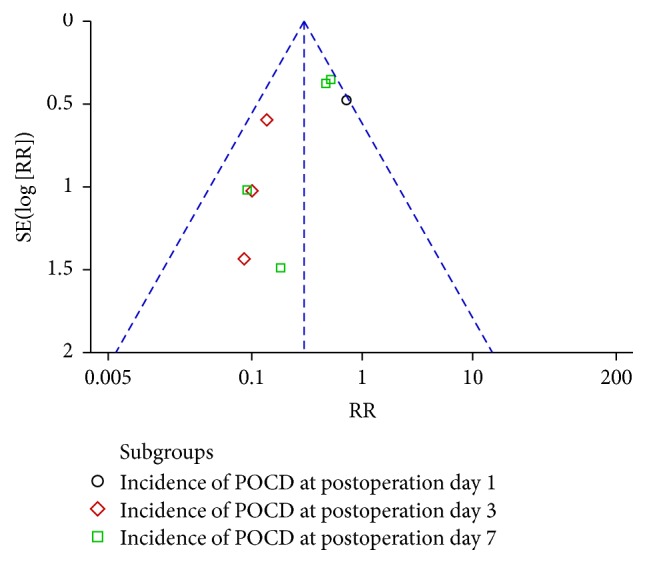
Funnel plot of ulinastatin versus control: incidence of POCD.

**Figure 7:**
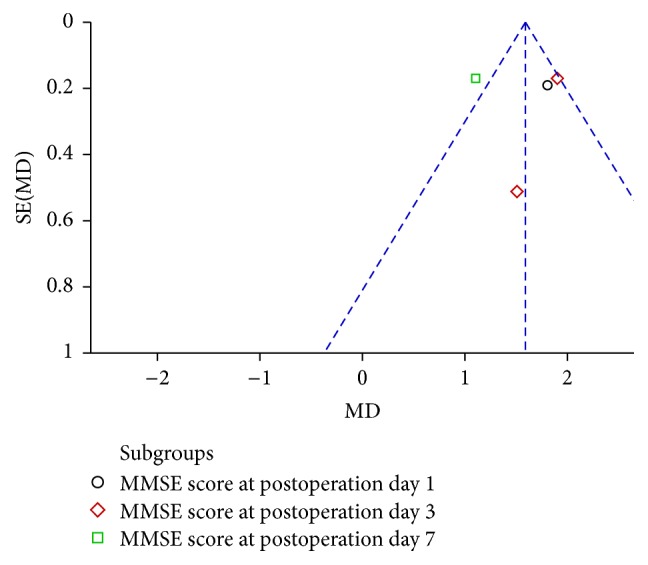
Funnel plot of ulinastatin versus control: MMSE score.

Publication bias was explored via funnel plots (Figures 6 and 7). Both funnel plots presented asymmetry, indicating publication bias.

## 4. Discussion

To the best of our knowledge, this is the first systematic review and meta-analysis to assess the clinical effect of ulinastatin in the treatment of patients with early POCD. Five RCTs involving a total of 461 patients were identified by our current work. Based on the findings of this study, ulinastatin administration was effective in reducing the incidence of POCD and improving MMSE score. Compared with control treatment, incidences of all types of postoperative complications were lower in ulinastatin groups. However, the effect of ulinastatin in reducing proinflammatory cytokines (IL-6 and TNF-*α*), CRP, and S100*β* remained to be further elucidated.

POCD refers to a deterioration in cognition noted to occur after surgery and anesthesia. It is a subtle form of cognitive decline that can occur after surgery and affect cognitive performance, especially in the elderly, although few young patients have been described as cognitive declines as well [31]. Surgical trauma can increase level of inflammatory cytokine IL-6, TNF-*α*, and level of CRP. These factors with a wide bioactivity can cross blood brain barrier, promote brain cell permeability, and cause an inflammatory reaction in the central nervous system, thereby affecting the functioning of synaptic connections, resulting in damage in cognitive function. Our included studies observed a significant increase of IL-6 after operation, and the concentration of IL-6 returned to normal on the third day after operation. The incidence of POCD and concentration of IL-6 were significantly lower than control within the first two days after operation; this suggested that ulinastatin may attenuate POCD by inhibiting the release of IL-6. Regarding the changes of TNF-*α* and CRP, the results among studies were inconsistent. Ge and colleagues [28] found that TNF-*α* were increased on postoperation day 1; both high dose and low dose administration of ulinastatin could inhibit the release of TNF-*α*. Xu et al. [26] did not find a postoperative increase of TNF-*α* and CRP; they attributed this to the populations differences.

S100*β* is a calcium-binding protein, mainly in stellate cells and Schwann cells, as glial marker protein, brain-specific proteins. After surgical operation, increased serum S100*β* values were reported to correlate with poor neurological outcome [26]. S100*β* was usually elevated in the blood and cerebrospinal fluid following nervous system damage due to a functional disturbance of membrane integrity and/or increased permeability of the blood brain barrier. Serum S100*β* concentrations were reported to be significantly increased within two days after operations, and it fell back to normal on the third day following operations. Xu et al. and Kang et al. [25, 26] found that ulinastatin could further reduce the concentration of S100*β* in serum than control in the first two days after operation. However, Ge and coworkers [28] found that S100*β* was significantly lower in ulinastatin group only on the postoperative 6 hours; no statistically significant difference of S100*β* was observed on other time-points studied. The biological half-life of S100*β* is approximately 30 minutes. Therefore, persistently increased levels of S100*β* in the blood indicate continuous release of this protein from damaged tissue. The discrepancies of postoperative S100*β* among our included studies can be explained by inconsistent method of ulinastatin administration and duration of operations.

MMSE is a 30-point scale that measures global cognitive function, with higher scores indicating better function, with scores <24 suggestive of cognitive impairment. Two studies measured MMSE score on postoperative day 1, day 3, and day 7. The findings suggested that ulinastatin was effective in improving the postoperative MMSE scores. In a word, ulinastatin could attenuate POCD, but whether ulinastatin played a pivotal part in reducing inflammatory mediators remained unclear, and the underlying mechanisms need to be validated by additional studies.

There were several limitations in our systematic review. Firstly, only five RCTs involving 461 patients that underwent surgical operations were selected by us; the sample-sizes of these studies were relatively small. Four studies did not mention blinding procedures, which might lead to exaggeration of conclusions drawn by these trials. Secondly, the MMSE remains one of the most commonly used instruments to assess cognitive outcomes, but it has been criticized for some shortcomings such as being less sensitive to milder cognitive impairments in older adults. Thirdly, all the neuropsychological tests and laboratory indicators were detected within a week after operations; the long-term benefits of ulinastatin on cognitive impairments could not be confirmed. Only one study reported the incidence of POCD one month after operation (high dose ulinastatin 12.9%, low dose ulinastatin 16.7%, and control 28.1%). Future studies with longer follow-ups to validate the beneficial effect of ulinastatin are needed. Besides, only two included studies reported duration of operations; considering that some inflammatory mediators were directly associated with duration of operations, the results and conclusions may be sequentially confounded. Lastly, the funnel plots indicated obvious publication bias; this could be explained by the fact that all these trials were conducted in China and all these articles were published in Chinese academic journals; these works have only been performed in a Chinese population, clinical studies within western culture to evaluate the effect of ulinastatin are encouraged.

## 5. Conclusion

In summary, ulinastatin administration showed remarkable effect in reducing the incidence of POCD and improving MMSE score. Ulinastatin could significantly reduce the concentrations of IL-6 and S100*β* from the end of operation to postoperative day 2; results regarding the changes in CRP and TNF-*α* remained debatable. Further studies are need not only to determine the potential benefit but to understand the mechanisms involved in the mitigation of POCD by ulinastatin.

## Figures and Tables

**Table 1:** Characteristics of included studies.

Study	Study design	Population	Intervention and control	Outcomes
Xu et al., 2013 China [26]	Prospective, double-blind, 2-arm RCT	Abdominal surgery under intravenous general anesthesia; U: 40 patients, 75.6 ± 7.2 years; C: 40 patients, 74.1 ± 8.1 years	U: ulinastatin 10000 units/kg diluted in normal saline to a volume of 100 mL (i.v.), over a period of 30 min before surgical incision and 5000 units/kg after surgery on days 1–3; C: 100 mL normal saline (i.v.)	Incidence of POCD; IL-6, TNF-*α*, CRP, S100*β*

Ge et al., 2011 China [24]	2-arm RCT	Hip joint replacement under combined spinal-epidural anesthesia; U: 80 patients, 72.8 ± 7.5 years; C: 80 patients, 75.0 ± 7.1 years	U: ulinastatin 10000 units/kg diluted in normal saline to a volume of 50 mL (i.v.), over a period of 30 min before surgical incision and 5000 units/kg after surgery on days 1–3; C: 50 mL normal saline (i.v.)	Incidence of POCD

Kang et al., 2010 China [25]	2-arm RCT	Hip joint replacement under combined spinal-epidural anesthesia; U: 40 patients, 75.0 ± 7.81 years; C: 40 patients, 72.8 ± 7.25 years	U: ulinastatin 10000 units/kg diluted in normal saline to a volume of 50 mL (i.v.), over a period of 30 min before surgical incision and 5000 units/kg after surgery on days 1–3; C: 50 mL normal saline (i.v.)	Incidence of POCD, MMSE score; S100*β*

Shan et al., 2015 China [27]	2-arm RCT	Hip fracture under combined spinal-epidural anesthesia; U: 21 patients, 78 ± 2 years; C: 27 patients, 75 ± 1 years	U: ulinastatin 5000 units/kg diluted in normal saline to a volume of 100 mL (i.v.), before surgical incision and 5000 units/kg immediately after surgery; C: 100 mL normal saline (i.v.)	Incidence of POCD, MMSE score; CRP

Ge et al., 2015 China [28]	3-arm RCT	Coronary artery bypass grafting under intravenous general anesthesia; U1: 31 patients, 69.1 ± 4.8 years; U2: 30 patients, 68.9 ± 4.7 years; C: 32 patients, 67.7 ± 5.4 years	U1: ulinastatin 16000 units/kg diluted in normal saline to a volume of 60 mL (i.v.) before anesthesia induction; U2: ulinastatin 8000 units/kg diluted in normal saline to a volume of 60 mL (i.v.) before anesthesia induction; C: 60 mL normal saline (i.v.)	Incidence of POCD; IL-6, TNF-*α*, CRP, S100*β*

Note: POCD: postoperative cognitive dysfunction; U: ulinastatin group; C: control group; i.v.: intravenously; RCT: randomized controlled trials.

**Table 2:** Secondary outcomes reported by included studies.

Study	IL-6 (pg/mL)		TNF-*α* (pg/mL)		CRP (mg/L)		S100*β* (ug/L)	
	U	C	U	C	U	C	U	C
Xu et al., 2013 [26]	7.1 ± 0.1^a^	8.2 ± 0.2^a^	870 ± 490^a^	890 ± 590^a^	6.8 ± 3.2^a^	7.4 ± 3.4^a^	0.039 ± 0.012^a^	0.040 ± 0.011^a^
	55.2 ± 5.1^b*∗*#^	98.3 ± 4.4^b*∗*^	1210 ± 450^b^	1380 ± 860^b^	108.3 ± 4.5^b*∗*^	109.8 ± 5.3^b*∗*^	0.097 ± 0.014^b*∗*#^	0.129 ± 0.034^b*∗*^
	46.2 ± 4.8^e*∗*#^	72.2 ± 3.8^e*∗*^	1070 ± 540^e^	1190 ± 750^e^	78.6 ± 3.6^e*∗*^	85.7 ± 5.1^e*∗*^	0.086 ± 0.016^e*∗*#^	0.141 ± 0.029^e*∗*^
	21.4 ± 7.3^f*∗*#^	45.3 ± 6.3^f*∗*^	950 ± 510^f^	960 ± 460^f^	54.6 ± 5.1^f*∗*^	65.3 ± 3.6^f*∗*^	0.057 ± 0.019^f*∗*#^	0.089 ± 0.038^f*∗*^
	8.2 ± 0.3^g^	9.3 ± 0.4^g^	880 ± 490^g^	890 ± 510^g^	7.4 ± 4.1^g^	8.9 ± 4.3^g^	0.042 ± 0.017^g^	0.047 ± 0.018^g^

Kang et al., 2010 [25]	—	—	—	—	—	—	0.040 ± 0.013^a^	0.041 ± 0.012^a^
							0.095 ± 0.021^b*∗*#^	0.125 ± 0.031^b*∗*^
							0.116 ± 0.017^c*∗*#^	0.178 ± 0.036^c*∗*^
							0.087 ± 0.019^e*∗*#^	0.142 ± 0.038^e*∗*^
							0.043 ± 0.012^g^	0.048 ± 0.015^g^

Shan et al., 2015 [27]	—	—	—	—	26 ± 5^a^	32 ± 5^a^	—	—
					64 ± 10^g*∗*#^	124 ± 7^g*∗*^		

Ge et al., 2015 (1) [28]	36.10 ± 5.48^a^	34.92 ± 4.68^a^	29.67 ± 4.17^a^	30.84 ± 3.98^a^	—	—	250 ± 30^a^	250 ± 40^a^
	49.66 ± 5.89^b*∗*#^	62.90 ± 7.23^b*∗*^	37.93 ± 6.80^b*∗*#^	44.09 ± 11.35^b*∗*^			770 ± 180^b*∗*^	810 ± 230^b*∗*^
	65.14 ± 10.86^d*∗*#^	90.63 ± 12.06^d*∗*^	51.92 ± 6.39^d*∗*#^	71.26 ± 11.33^d*∗*^			620 ± 160^d*∗*#^	770 ± 210^d*∗*^
	48.03 ± 6.01^e*∗*#^	61.20 ± 6.17^e*∗*^	62.55 ± 12.07^e*∗*#^	80.98 ± 15.33^e*∗*^			430 ± 90^e*∗*^	470 ± 100^e*∗*^

Ge et al., 2015 (2) [28]	34.67 ± 4.77^a^	34.92 ± 4.68^a^	30.24 ± 4.05^a^	30.84 ± 3.98^a^	—	—	240 ± 50^a^	250 ± 40^a^
	48.56 ± 6.25^b*∗*#^	62.90 ± 7.23^b*∗*^	38.17 ± 5.70^b*∗*#^	44.09 ± 11.35^b*∗*^			750 ± 170^b*∗*^	810 ± 230^b*∗*^
	68.16 ± 9.05^d*∗*#^	90.63 ± 12.06^d*∗*^	50.42 ± 3.27^d*∗*#^	71.26 ± 11.33^d*∗*^			590 ± 180^d*∗*#^	770 ± 210^d*∗*^
	47.02 ± 6.73^e*∗*#^	61.20 ± 6.17^e*∗*^	61.30 ± 11.91^e*∗*#^	80.98 ± 15.33^e*∗*^			440 ± 100^e*∗*^	470 ± 100^e*∗*^

Note: U: ulinastatin group; C: control group; ^a^preoperative; ^b^at the end of operation; ^c^three hours after operation; ^d^six hours after operation; ^e^one day after operation; ^f^two days after operation; ^g^three days after operation; ^*∗*^ *P* < 0.05 from preoperation in group U and group C (statistically significant); ^#^ *P* < 0.05 from group C (statistically significant). Ge et al., 2015 (1): high-dose ulinastatin (16000 U/kg); Ge et al., 2015 (2): low-dose ulinastatin (8000 U/kg).
